# (3Z)-5-Chloro-3-(Hydroxyimino)indolin-2-one attenuates hyperglycemia, increased hepatic glycogen content and hepatic damage induced by malathion acute exposure in rats

**DOI:** 10.1186/s12986-019-0374-3

**Published:** 2019-09-05

**Authors:** Edina da Luz Abreu, Anne Suély Pinto Savall, Allyson Ardais Boneberg, Bianca Barreto Martins, Vanessa Carratú Gervini, Tuane Bazanella Sampaio, André Ricardo Fajardo, Natália Paroul, Daniel Henrique Roos, Simone Pinton

**Affiliations:** 1 Universidade Federal do Pampa (UNIPAMPA) Campus Uruguaiana, Uruguaiana, RS CEP 97500-970 Brazil; 2 Universidade Federal do Rio Grande - Campus Carreiros, Rio Grande, RS CEP 96201-900 Brazil; 3 Instituto Federal de Educação, Ciência e Tecnologia Farroupilha – Campus Santo Ângelo, Santo Ângelo, RS CEP 98806-700 Brazil; 4 Universidade Federal de Pelotas (UFPel) - Campus Capão do Leão s/n, Pelotas, RS CEP 96010-900 Brazil; 5 Universidade Regional Integrada (URI), Campus Erechim, Erechim, RS CEP 99709-910 Brazil

**Keywords:** Cholinesterases, Aminotransferases, Antioxidant enzymes, Glucose metabolism, Organophosphate, Oxime

## Abstract

**Background:**

Organophosphorus pesticides (OP’s) are heavily constituted in agriculture, gardens, home and veterinary and although it is useful, there are concerns about the environment, safety and health of human and animals. In this study, we investigated the effects of a new oxime, (3Z)-5-Chloro-3-(Hydroxyimino)indolin-2-one (OXIME) against the alterations induced by malathion, an OP insecticide, acute exposure on markers of hepatic damage, glucose homeostasis, oxidative stress in rats cholinesterase (ChE) activity in rats.

**Methods:**

Adult male Wistar rats were divided into four groups: Control; Malathion; OXIME; and Malathion+OXIME. Twelve hours after co-treatment with malathion (250 mg/kg, i.p.) and/or OXIME (50 mg/kg, i.g.), the plasma and liver samples were collected for biochemical analyses.

**Results:**

The OXIME blocked the increase of plasma markers of hepatic function (AST and ALP) and the enzymatic inhibition of catalase and glutathione reductase in the liver of malathion-treated rats. Moreover, the hepatic cholinesterases inhibition induced by malathion acute exposure was suppressed by OXIME treatment. As assessed, a single dose of OXIME lowered the glycemia levels and hepatic glycogen content enhanced by malathion.

**Conclusions:**

This study suggests promise effects of (3Z)-5-Chloro-3-(Hydroxyimino) indolin-2-one against the hyperglycemia and the hepatic damage induced by malathion acute exposure, as well as its use as a ChE activity reactivator.

## Introduction

The organophosphates pesticides (OP) are chemicals compounds widely used in agriculture, gardens, household and veterinary [1]. Among the OP, malathion [S-1,2(bis-ethoxycarbonyl)ethyl-O,O-dimethyl phosphorodithioate] stands out due to its high toxicity [2, 3]. Malathion is distributed mainly to the liver, kidney, small intestine, urinary tract, and lungs. The bioactivation of malathion is mediated mainly by enzymes of cytochrome P450 in the liver [4], generating the active metabolite malaoxon [5, 6].

The OP’s are associated with adverse effects on human and animal health [7–9]. Theirs primary mechanism of toxicity involve the inhibition of acetylcholinesterase (AChE) activity. Besides the cholinergic system, the OP-exposure can trigger several physiological responses including alterations in the glucose homeostasis [10–13], hyperglycemia [12, 14, 15] and oxidative stress [16–20]. Further, the exposition to OP alters biochemical parameters of hepatotoxicity [6, 16, 21, 22], indicating the occurrence of liver injury [12, 21, 23].

Glucose homeostasis changes and hepatic damage have been largely investigated and indicate the involvement of additional toxicity pathways of OP. In fact, changes in the glucose homeostasis have been demonstrated both in animals subjected to acute and chronic exposure to OP’s [10–13] as in OP-exposed humans [24, 25]. In this way, glycogenolysis, gluconeogenesis, and hypothalamus-pituitary-adrenal (HPA) axis seem to be affected by OP exposure resulting in hyperglycemia [12, 14].

In regards to oxidative stress, studies demonstrated that the exposure to OP resulted in increased lipid peroxidation and decreased glutathione levels in liver, kidney, heart, blood and brain structures [17, 18, 21]. In addition, the activities of the antioxidant enzymes glutathione peroxidase (GPx), glutathione reductase (GR), superoxide dismutase (SOD) and catalase (CAT) also were altered by OP in several tissues [16–18].

The standard therapeutic strategy for acute exposure to OP includes the use of anticholinergics (muscarinic receptor antagonists), AChE reactivators drugs (oximes), and diazepam [26]. Oxime derivatives (e.g. pyridinium oximes) have been used as an antidote in the detoxification step. However, there is no compelling evidence about the efficacy of oximes therapies since the results have shown that the success of these therapies depends both oxime and OP structure-activity [26–28].

**Fig. 1:**
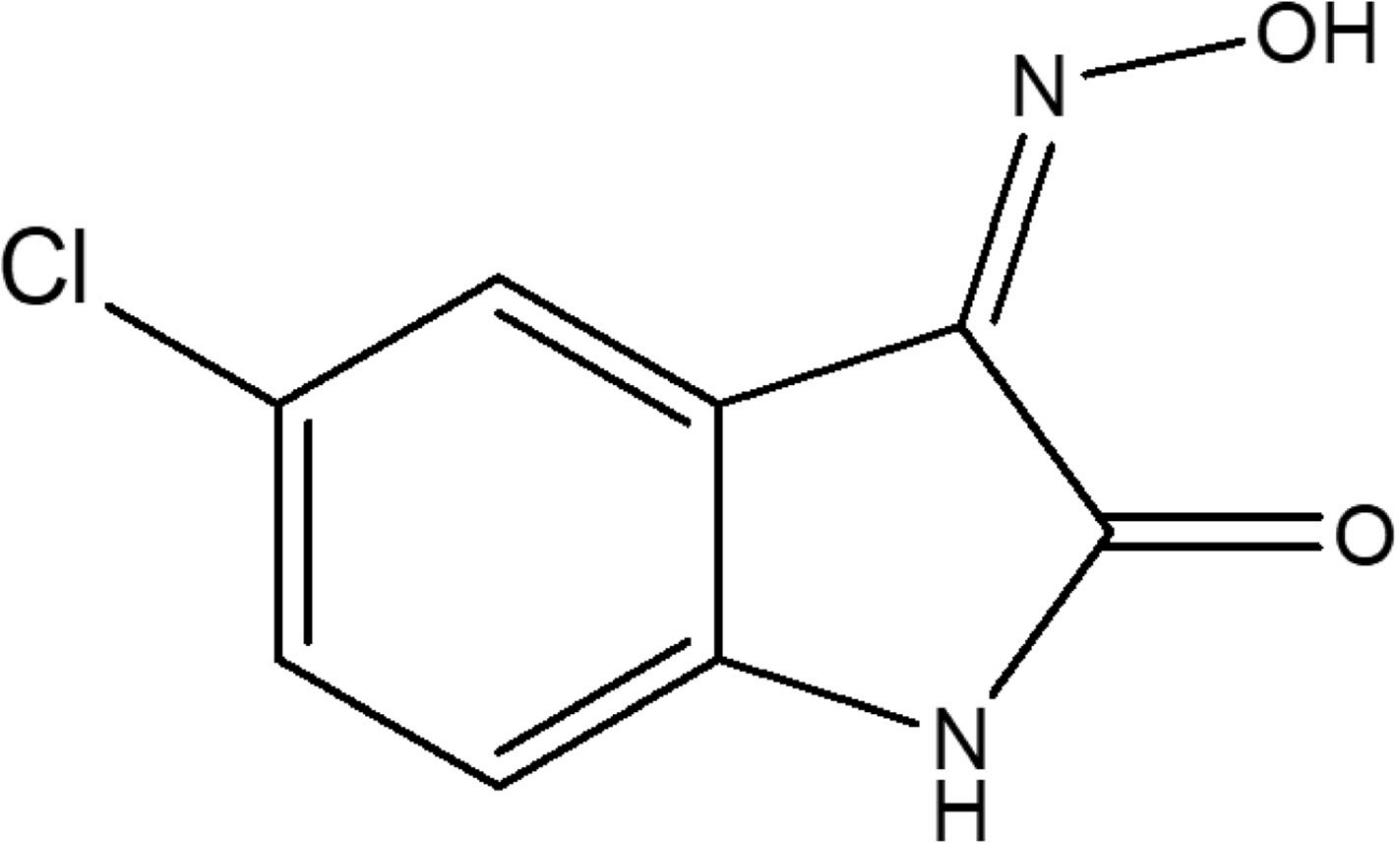
Chemical structure of (3Z)-5-Chloro-3-(Hydroxyimino)indolin-2-one (OXIME)

Based on these findings, new drugs or alternative approaches have been considered for OP poisoning therapy [27, 29]. The (3Z)-5-Chloro-3-(Hydroxyimino)indolin-2-one (Fig. 1) (OXIME) is a novel oxime derived from isatin (1*H*-indole-2,3-dione). Since isatin and its derivatives show various biological activities [20, 30] it is plausible that OXIME might be useful against OP poisoning.

Therefore, the purpose of this study was investigating the effectiveness of a single dose of OXIME against the alterations induced by malathion acute exposure on markers of hepatic damage, glucose homeostasis and oxidative stress in rats. Additionally, we also addressed the ability of the OXIME reactivate the cholinesterase (ChE) activity, reducing the adverse effects of malathion.

## Materials and methods

### Drugs and chemicals

Malathion 500 CE (5.0%) (Biocarb Chemical Industry LTDA, Curitiba, PR, Brazil) was obtained from commercial grade. (3Z)-5-Chloro-3-(Hydroxyimino)indolin-2-one (OXIME) was synthesized at the School of Chemistry and Food of Federal University of Rio Grande (FURG), RS, Brazil as described [31]. For this, an equimolar mixture of 5-chloroisatin and hydroxylamine chlorhydrate in ethanol was used. The reaction medium was acidified using acetic acid and placed under reflux (135 °C) for 5 h. The reaction was vacuum filtered and the compound was isolated as a golden yellow precipitate. After that, the isolated compound was washed in cold distilled water (F.P. 252–274 °C). Analysis of the ^1^H ^13^C nuclear magnetic resonance spectroscopy analysis showed that the compound obtained presented analytical and spectroscopic data in full agreement with its assigned structure. The chemical purity of OXIME (99.9%) was determined by gas chromatography–mass spectrometry. All other chemicals were obtained from analytical grade and standard commercial suppliers.

### Animals

The experiments were carried out using male adult Wistar rats (*n* = 28), 2 months of age (250–300 g), purchased from Federal University of Santa Maria, RS, Brazil. The animals were provided with food (commercial diet) and water ad libitum and maintained in the animal house at controlled conditions: temperature (22 ± 2 °C) and 12 h light-dark cycle (lights on at 7:00 a.m.). All experiments were performed according to the local ethics committee of the Federal University of Pampa, Brazil (CEUA/UNIPAMPA 024/2016). All efforts were made to minimize animals suffering and to reduce the number of animals used in the experiments.

### Experimental design

In order to access the effects of an acute exposure to malathion and the putative beneficial effects of OXIME on markers of hepatic damage, glucose homeostasis, oxidant-antioxidant system and ChE activity, rats were randomized into four groups with seven animals per group:

I. **Control** - distilled water by intraperitoneal injection (i.p.; 5 mL/kg) plus canola oil by intragastric gavage (i.g.; 3 mL/kg);
II. **Malathion** - malathion (250 mg/kg; i.p.) plus canola oil (i.g);
III. **OXIME** - distilled water (i.p.) plus [(3Z)-5-Chloro-3-(Hydroxyimino) indolin-2-one] (50 mg/kg; i.g.);
IV. **Malathion + OXIME** - malathion (250 mg/kg; i.p.) plus [(3Z)-5-Chloro-3-(Hydroxyimino) indolin-2-one] (50 mg/kg; i.g.);

The malathion dose of 250 mg/kg was selected based on a previously published study [32], which demonstrated that malathion caused metabolic disorders in rats. The OXIME dose of 50 mg/kg was established through a pilot study by our research group (data not shown).

Twelve hours after treatment with malathion and/or OXIME and/or vehicles, following overnight fasting, all rats were anesthetized for blood collection by heart puncture. The time to collect of samples (12 h) was based on a pilot study of our research group and other results described [33, 34]. Plasma was extracted by centrifugation at 2500 *g* for 10 min and conserved at − 20 °C for posterior analyses. In the sequence, the animals were killed and their livers were removed. Except for protein carbonyl content assay and ChE activity, the liver samples were homogenized in 50 mM Tris-HCl (pH 7.4; 1:10 *w/v*) and centrifuged at 2500 *g* for 10 min at 4 °C. The low-speed supernatants (S_1_) were used to the biochemical assays.

### Glucose homeostasis evaluation

Blood glucose levels and hepatic glycogen content were measured as indicators of the glucose homeostasis. Blood glucose levels were established by an enzymatic method based on the oxidase/peroxidase system using a commercial kit (Bioclin, Belo Horizonte, Minas Gerais, Brazil). Blood glucose levels results were expressed in mg/dL. Hepatic glycogen content was performed according to the method described [35]. Briefly, 0.3 g of the liver was digested in 3 mL of KOH 30%, incubated for 10 min at 90 °C. After that, glycogen was precipitated with 2 mL of ethanol and resuspended in 0.2 mL 5 M HCl and 0.8 mL distilled water. The glycogen content was measured with iodine reagent at 460 nm and expressed as % of hepatic glycogen.

### Hepatic function markers

Plasma samples were used to determine the aspartate aminotransferase (AST), alanine aminotransferase (ALT), alkaline phosphatase (ALP) and lactate dehydrogenase (LDH) activities as a parameter of the hepatic function. The colorimetric method was carried out to measure both AST and ALT activities [36]. Whereas, ALP and LDH activities were accessed using a commercial kit (Bioclin, Belo Horizonte, Minas Gerais, Brazil). The values were expressed as U/dL.

### Oxidative stress markers

#### Thiobarbituric acid reactive species (TBARS) levels

Hepatic TBARS levels, a measure of lipid peroxidation, were performed using an aliquot (200 μL) of S_1_, 500 μL thiobarbituric acid (0.8%), 500 μL acetic acid buffer, 200 μL sodium dodecyl sulfate (SDS, 8.1%) and 100 μL distilled water. The mixture was incubated at 95 °C for 2 h. The absorbance was measured at 532 nm. The results were expressed as nmol MDA/mg protein as described [37].

#### Protein carbonyl content

Protein carbonyl content in the liver was determined through of the reaction between protein carbonyls and 2,4-dinitrophenylhydrazine forming dinitrophenylhydrazone, a yellow compound [38]. Briefly, hepatic homogenates without centrifugation were diluted 1:10 (*v/v*) and an aliquot of 1 mL was added to the reaction mixture containing 200 μL of 10 mM dinitrophenylhydrazine (prepared in 2 M HCl). All tubes were incubated at room temperature in the dark for 1 h and shaken with a vortex mixer each 15 min. After that, 500 μL of denaturation buffer, 1.5 mL of ethanol and 1.5 mL of hexane were added to each tube. The tubes were shaken with a vortex mixer for 40 s and centrifuged at 3000 *g* for 15 min. The supernatants obtained were discarded. The pellets were washed twice with 1 mL ethanol: ethyl acetate (1:1, *v/v*) and resuspended in 1 mL of denaturation buffer. Absorbance was determined at 370 nm. Data were expressed as nmol carbonyl content/mg protein.

#### Non-protein sulfhydryl (NPSH) content

To determine hepatic NPSH content, S_1_ was mixed (1:1) with 10% trichloroacetic acid. After the centrifugation, the protein pellet was discarded and the free SH-groups were determined in the clear supernatant. An aliquot of supernatant was added in 1 M potassium phosphate buffer pH 7.4 and 10 mM DTNB (5,5′-dithiobis-2-nitrobenzoic acid). The color reaction was measured at 412 nm. NPSH levels were expressed as nmol NPSH/g tissue [39].

#### Enzymatic antioxidant defenses

Catalase (CAT) activity was spectrophotometrically assayed by the method [40], which involves monitoring the consumption of H_2_O_2_ in the S_1_ at 240 nm. The enzymatic reaction was initiated by adding an aliquot of 40 μL of the S_1_ from liver samples and the substrate (H_2_O_2_) to a concentration of 0.3 M in a medium containing 50 mM phosphate buffer, pH 7.0. The enzymatic activity was expressed in Units (1 U decomposes 1 μmol H_2_O_2_/min at pH 7 at 25 °C)/mg protein.

Glutathione peroxidase (GPx) activity in the liver was measured by the nicotinamide adenine dinucleotide phosphate (NADPH) oxidation rate at 340 nm using H_2_O_2_ as substrate [41]. The reaction mixture consisted of EDTA, NADPH, GSH, sodium azide, and glutathione reductase (GR). The reaction was initiated by the addition of H_2_O_2_. The disappearance of NADPH at 340 nm was recorded at room temperature. Enzyme activity was defined as nmol NADPH/min/mg protein.

GR activity in the liver was estimated by the method describe [42]. The reagent mixture was composed of 150 mM potassium phosphate buffer (pH 7.0), 1.5 mM EDTA, 0.15 mM NADPH. Oxidized glutathione (GSSG) was used as the substrate. GR activity is proportional to NADPH decay at 340 nm. The enzymatic activity was expressed as nmol NADPH/min/mg protein.

### Hepatic cholinesterase (ChE) activity

Samples of liver were homogenized in 0.25 M sucrose buffer (1:10, w/v) and centrifuged at 2400 *g* for 15 min at 4 °C. ChE activity (a mixture of butyrylcholinesterase and AChE) was carried out according to the method describe [43], using acetylthiocholine as substrate. The activity of ChE was spectrophotometrically measured at 412 nm and expressed as nmol/min/mg protein.

### Protein determination

The protein content was quantified by the Bradford [44] method using Coomassie blue. The mixture was incubated at room temperature for 10 min and the developed color was spectrophotometrically determined at 595 nm. Bovine serum albumin (1 mg/mL) was used as the standard.

### Statistical analysis

The normal distribution of the data was tested with D’Agostino and Pearson normality test. Statistical analysis was performed using a two-way analysis of variance (ANOVA) followed by the Tukey’s multiple range test when appropriated (GraphPad Prism 6 software, San Diego, CA, USA). Data were expressed as the mean ± standard error of mean (S.E.M.) of 7 animals/group. A value of *p* < 0.05 was considered significant.

## Results

### Glucose homeostasis determination

**Fig. 2:**
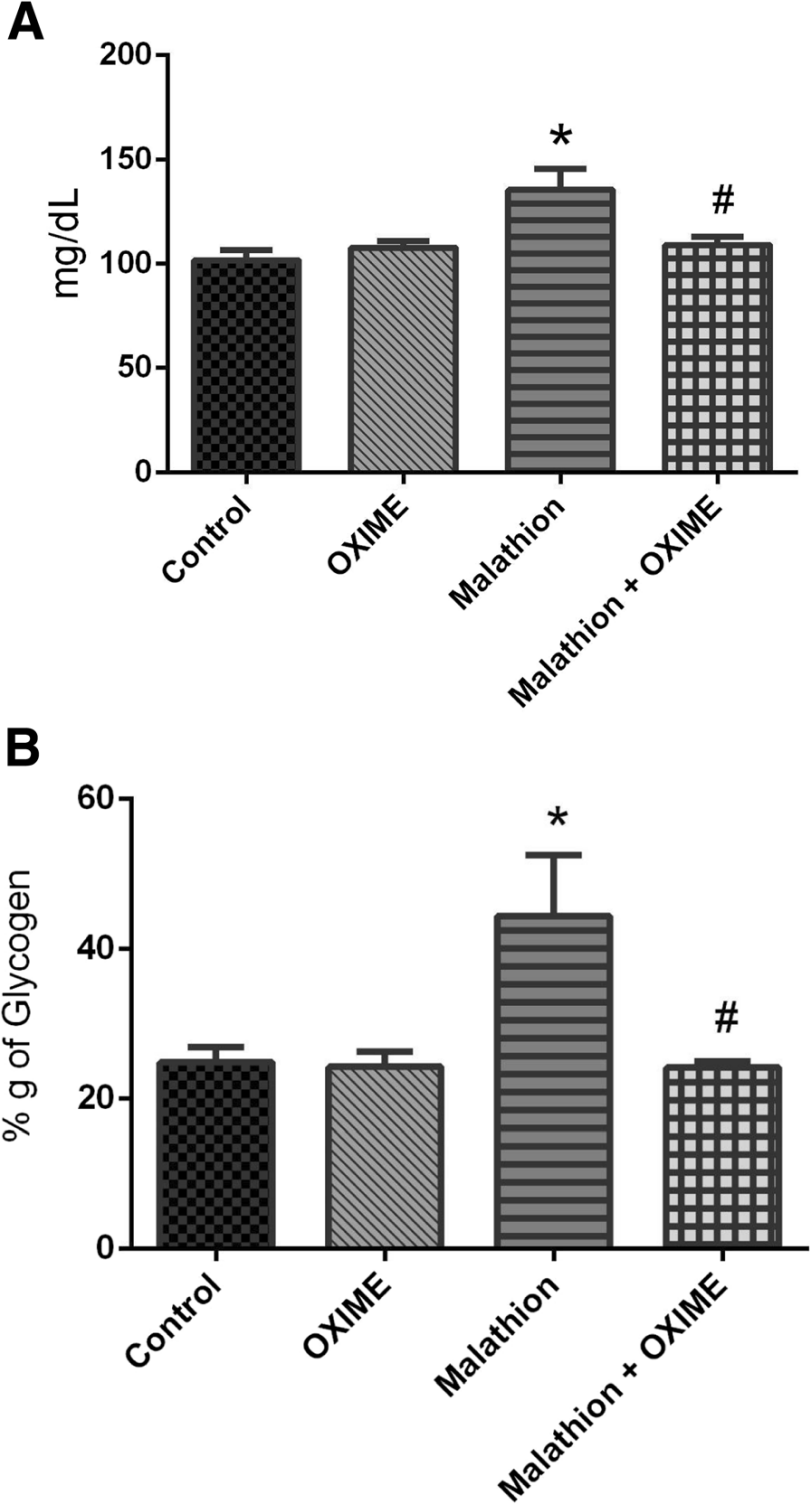
Effects of malathion and/or OXIME treatments on the levels of blood glucose (**a**) and hepatic glycogen (**b**) of rats. Data are reported as mean ± S.E.M. of 7 animals per group. *p < 0.05 as compared to the control group; ^#^*p* < 0.05 as compared to the malathion group (two-way ANOVA/Tukey’s test)

Blood glucose levels were substantially increased by malathion (around 35%) as compared to control group (F_1,24_ = 6.36, *p* = 0.0187), while that a single dose of OXIME prevented the hyperglycemia caused by malathion in rats (*p* = 0.0355) (Fig. 2a). Furthermore, two-way ANOVA of hepatic glycogen data showed a significant main effect of the malathion (F_1,24_ = 5.26, *p* = 0.0309) (Fig. 2b). Malathion significantly raised the glycogen contents in the liver of rats (*p* = 0.0174, around 77% when compared to control group) and the OXIME was able to block this effect (*p* = 0.0134, when compared to malathion group) (Fig. 2b).

### Hepatic function markers

Two-way ANOVA revealed a significant increase in ALT, AST and ALP activities in the plasma of rats exposed to malathion when compared to those of the control group (ALT [F_1,24_ = 7.27, *p* = 0.0126]; AST [F_1,24_ = 14.79, *p* = 0.0008]; ALP [F_1,24_ = 9.52, *p* = 0.0051]) (Table 1). Importantly, OXIME treatment was effective in reversing the AST (*p* = 0.0004) and ALP (*p* = 0.0438) activities increased by malathion, but it was not effective to restore the ALT activity in the plasma of rats exposed to malathion (Table 1). The LDH activity was not altered by the malathion and/or OXIME treatments.

**Table 1:** Effects of OXIME on ALT, AST, LDH, and ALP activities in the plasma of rats exposed to malathion

Group	ALT (U/dL)	AST (U/dL)	LDH (U/dL)	ALP (U/dL)
Control	43.69 ± 13.72	106.68 ± 12.95	393.20 ± 41.00	60.84 ± 16.67
OXIME	68.95 ± 21.35	115.78 ± 7.32	466.00 ± 59.40	119.76 ± 25.20
Malathion	172.83 ± 51.03*	201.01 ± 10.78*	373.60 ± 77.60	189.53 ± 39.96*
Malathion + OXIME	118.02 ± 26.95	117.39 ± 15.58^#^	395.40 ± 107.00	81.50 ± 20.34^#^

Data are reported as the mean ± S.E.M. of 7 animals per group and expressed as U/dL. *p < 0.05 as compared to the control group, ^#^*p* < 0.05 as compared to the malathion group (two-way ANOVA/Tukey’s multiple range test)

### Oxidative stress markers

As shown in the Table 2, malathion and/or OXIME treatments caused alteration neither in the TBARS (F_1,24_ = 0.0346, *p* = 0.8539) nor in protein carbonyl levels (F_1,24_ = 0.3150, *p* = 0.8798). In addition, there was no significant difference in the NPSH levels among groups (F_1,24_ = 0.8783, *p* = 0.3580) (Table 2).

**Table 2:** Effects of OXIME on TBARS, protein carbonyl and NPSH levels in the liver of rats exposed to malathion

Group	TBARS	Protein Carbonyl	NPSH
Control	12.99 ± 1.46	10.41 ± 0.32	1.31 ± 0.063
Malathion	12.38 ± 0.67	9.39 ± 0.61	1.36 ± 0.057
OXIME	14.45 ± 1.41	10.12 ± 0.45	1.23 ± 0.061
Malathion + OXIME	13.40 ± 1.02	9.58 ± 0.22	1.40 ± 0.072

Data are reported as the mean ± S.E.M. of 7 animals per group and expressed as nmol MDA/mg protein, nmol carbonyl content/mg protein and nmol NPSH/g tissue, respectively (two-way ANOVA)

### Enzymatic antioxidant defenses

**Fig. 3:**
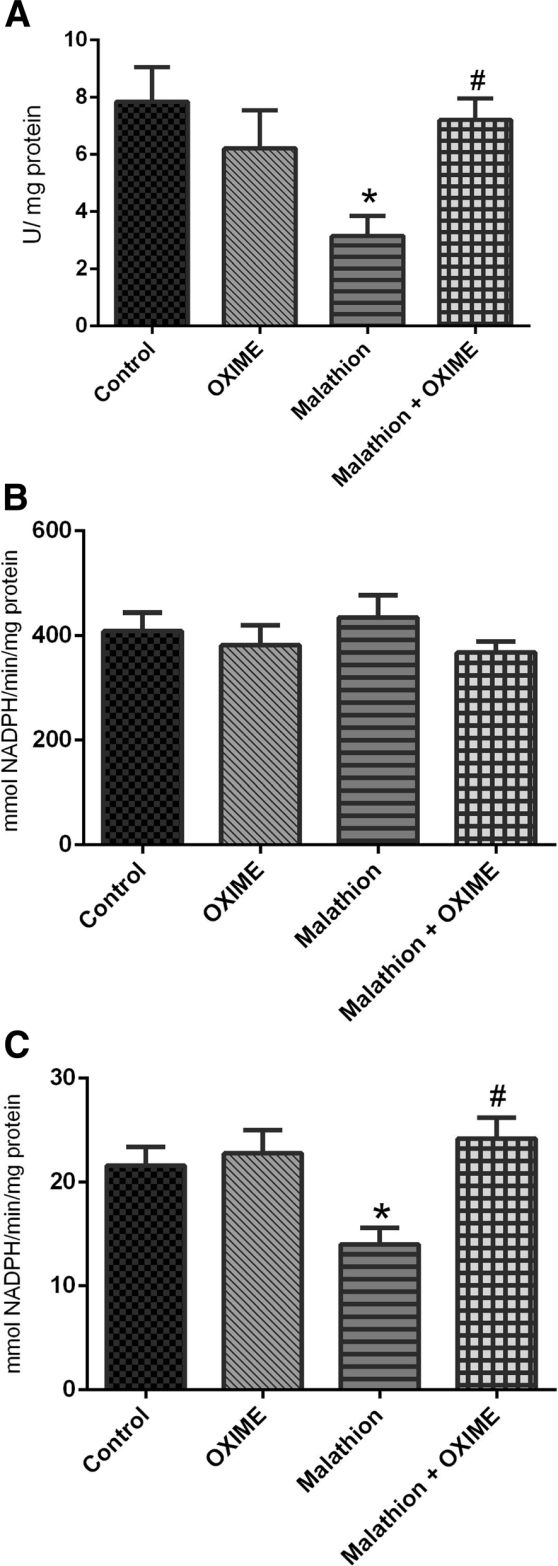
Effects of malathion and/or OXIME treatments on the catalase (**a**), glutathione peroxidase (**b**) and glutathione reductase (**c**) activities in the liver of rats. Data are reported as mean ± S.E.M. of 7 animals per group. *p < 0.05 as compared to the control group; ^#^*p* < 0.05, as compared to the malathion group (two-way ANOVA/Tukey’s test)

Two-way ANOVA of CAT activity demonstrated a significant malathion × OXIME interaction (F_1,24_ = 7.58, *p* = 0.0111) in liver of rats. As shown in the Fig. 3a, acute exposure to malathion decreased the CAT activity (around 60%) in the liver, which were protected by the OXIME treatment (*p* = 0.0495). However, there was no significant difference in the GPx activity among groups in liver of rats (Fig. 3b) (F_1,24_ = 0.3327, *p* = 0.5695). Moreover, a significant difference was founded in GR activity in the liver of rats (F_1,24_ = 5.22, *p* = 0.0315). Acute exposure to malathion decreased the GR activity (around 36%), which were protected by the OXIME treatment (*p* = 0.0067) (Fig. 3c).

### Hepatic ChE activity

**Fig. 4:**
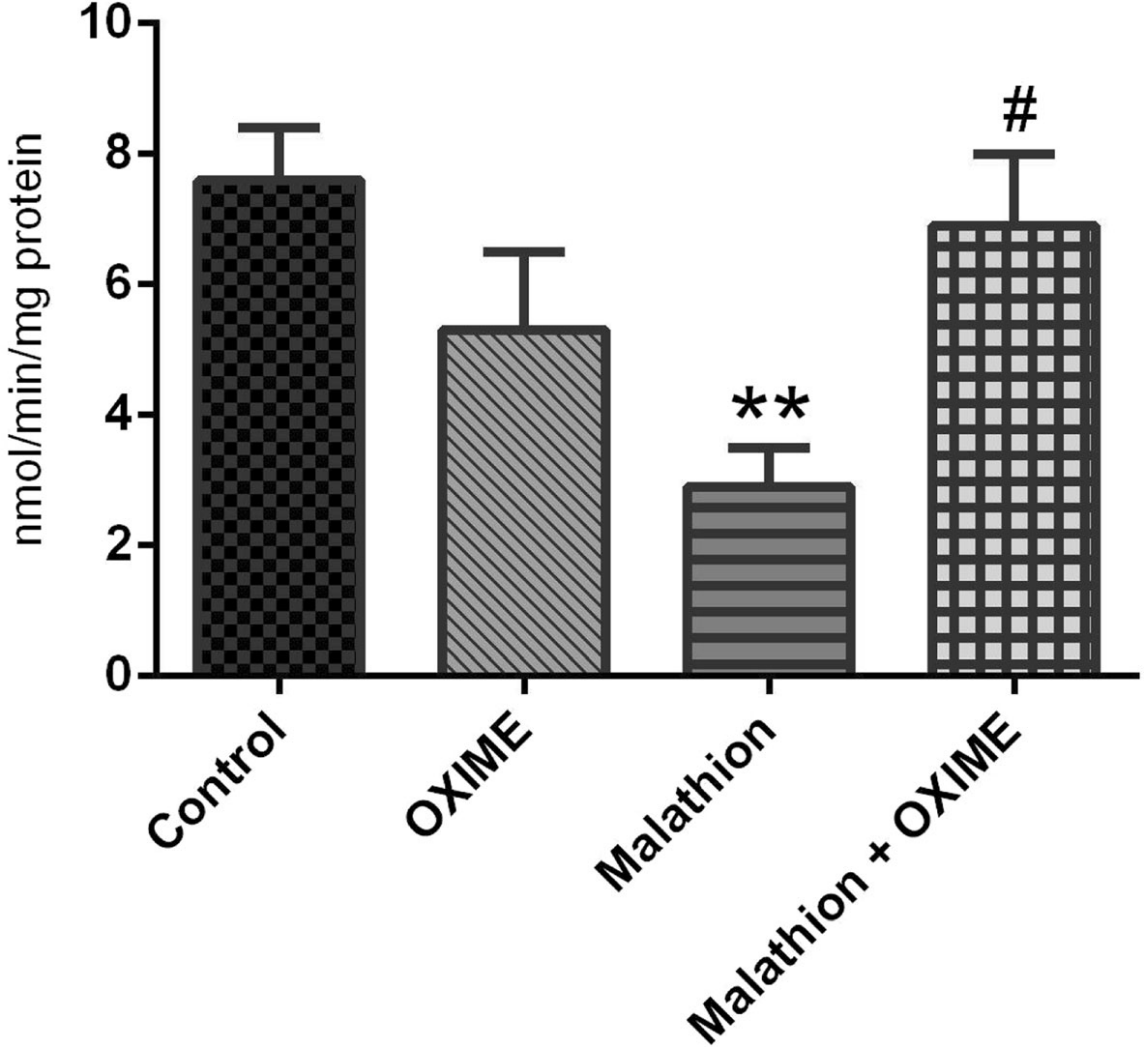
Effects of malathion and/or OXIME treatments on the hepatic ChE activity of rats. Data are reported as mean ± S.E.M. of 7 animals per group. **p < 0.01 as compared to the control group; ^#^*p* < 0.05 as compared to the malathion group (two-way ANOVA/Tukey’s test)

Data from hepatic ChE activity demonstrated a significant malathion × OXIME interaction (F_1,24_ = 11.59, *p* = 0.0023). Tukey’s post hoc test comparisons revealed that the malathion inhibited the ChE activity in the liver of rats when compared to those of the control group (around 63%). Moreover, a single dose of OXIME blocked the malathion-induced ChE inhibition (*p* = 0.0252) (Fig. 4).

## Discussion

The present findings demonstrate the beneficial effects of OXIME against to toxicity induced by malathion acute exposure in rats. A single dose oral of OXIME demonstrated a hypoglycemic action and was effective in reducing the increase of the hepatic glycogen content caused by malathion. Concerning the hepatoprotective analysis, the OXIME blocked the increase of the AST and ALP activities in the plasma and the inhibition of the CAT and GR activities in the liver of malathion-treated rats. Importantly, the hepatic ChE inhibition induced by malathion acute exposure was prevented by OXIME treatment.

The long application of OP in agricultural programs was accompanied by a potentially hazardous impact on humans, animals, plants, and the environment (water, air, soil, and food) and causes severe acute and chronic poisoning [45]. In fact, the toxicity of OP results in adverse effects on many organs and systems such as the liver, kidney, nervous system, immune system, and reproductive system [46].

Among the metabolic disorders induced by OP, the alterations in the glucose homeostasis have been highlighted [11, 12, 24, 25, 47]. In the present study, malathion elevated the blood glucose levels of the rats and this alteration was suppressed by the OXIME treatment. Indeed, it was demonstrated that a single dose of malathion [11, 34], as well as other Ops [14, 15, 33], is associated with increased blood glucose levels in rats. Studies suggest that the hyperglycemic effect of malathion is linked to stimulation of glycogenolysis, gluconeogenesis pathways and insulin resistance [22, 48].

Based on the stimulation of glycogenolysis and gluconeogenesis pathways, we expected a decrease in hepatic glycogen levels by malathion [22, 34]. However, we also observed an increase in the hepatic glycogen levels after the malathion acute exposure, similar results have been reported in rats after acute administration of malathion [49] and chlorpyrifos [50]. Reported [51] that the increase of glycogen levels in the liver is due to stimulation of the glycogen synthetase activity, a key enzyme of glycogenesis. The treatment with a single dose of OXIME was also able to prevent the increase in the hepatic glycogen levels of the rats exposed to malathion.

The liver is one of the main target organs of the malathion toxicity. This OP causes degenerative changes and disrupts the hepatic architecture [23]. Hepatocellular necrosis is commonly investigated through the determination of plasma activities of aminotransferases AST and ALT, that are localized in the hepatocytes [52]. In this way, our results corroborate with the literature that show increased levels of AST, ALT, and ALP activities after malathion exposure [16, 51]. In addition, we demonstrated that a single dose of OXIME modulated the activities of AST and ALP, evidencing its - at least in part - hepatoprotective effect.

Surprisingly, our data pointed out no change in the levels of TBARS, NPSH and protein carbonyl in the liver of rats exposed to malathion. Although the oxidative stress is an important component to the mechanism of malathion toxicity [1, 17, 53], it should be noted that probably the schedule of protocol exposure was the accountable for the lack of alteration in these parameters.

On the other hand, the malathion acute exposure induced a decrease in the GR and CAT activities in the liver, which was suppressed by a single dose of OXIME. Glutathione system represents the main antioxidant defense. Glutathione has a crucial role in cellular signaling and antioxidant defenses either by reacting directly with reactive oxygen or nitrogen species. In concert with its dependent enzymes, denoted as the glutathione system, glutathione is responsible for the detoxification of reactive oxygen and nitrogen species (ROS/RNS) and electrophiles produced by xenobiotics [54].

Currently, the treatment with oximes is established for malathion poisoning. However, the oximes clinically available display many side effects, such as low penetration into the blood-brain barrier and inefficient nucleophilic action [55]. In this context, there is a clear demand for new reactivators of malathion-inhibited ChE activity with a higher efficacy than those available. Of note, the OXIME tested in the present study showed a remarkable effect on the ChE activity inhibition caused by malathion acute exposure, indicating its potential as a ChE activity reactivator in the malathion poisoning. Finally, the inhibition of the ChE is of marked interest in OP toxicity, because it may be the route of activation of the HPA axis and consequently to stimulate gluconeogenesis [14].

## Conclusion

In summary, acute exposure to malathion induced changes in the glycemia and glycogen metabolism, hepatotoxicity, reduced activities of enzymatic antioxidant defenses and ChE in the liver. Importantly, a single dose of (3Z)-5-Chloro-3-(Hydroxyimino)indolin-2-one attenuated the malathion-induced toxicity in rats. Malathion exposure has been associated with metabolic disorders, thus, this study suggests promise effects of (3Z)-5-Chloro-3-(Hydroxyimino) indolin-2-one against the hyperglycemia and the hepatic damage induced by malathion acute exposure, as well as its use as a ChE activity reactivator. However, more studies are necessary to elucidate its mechanisms of action.

## Data Availability

The datasets used and/or analysed during the current study are available from the corresponding author on reasonable request.

## Abbreviations

AChE: Acetylcholinesterase
ALP: Phosphatase alkaline
ALT: Alanine aminotransferase
ANOVA: Analysis of Variance
ANVISA: National Health Surveillance Agency
AST: Aspartate aminotransferase
CAT: Catalase
CEUA: Ethics Committee Use Animals of UNIPAMPA
ChE: Cholinesterase
CNS: Central Nervous System
DTNB: 5,5′-dithiobis-2-nitrobenzoic acid
EDTA: Ethylenediamine tetra acetic acid
GPx: Glutathione Peroxidase
GR: Glutathione Reductase
GSSG: Oxidized glutathione
HPA: Hypothalamus-pituitary-adrenal
i.g: Intragastric
i.p: Intraperitoneal
LDH: Lactate dehydrogenase
MAPA: Ministry of Agriculture Livestock and Food Supply
NADPH: Nicotinamide adenine dinucleotide phosphate
NPSH: Non-protein thieves.
OP: Organophosphorus.
TBARS: Thiobarbituric acid reactive species
